# 9-(2-Bromo­eth­yl)-9*H*-carbazole

**DOI:** 10.1107/S1600536812024397

**Published:** 2012-06-13

**Authors:** Bao-Hua Zhao, Xiao-Fei Zhu, Shuang Guan, Dong-Feng Li

**Affiliations:** a School of Chemistry and Life Science, Changchun University of Technology, Changchun 130012, People’s Republic of China

## Abstract

In the title compound, C_14_H_12_BrN, the fused-ring system is slightly buckled as its two benzene rings are inclined to one another by 3.41 (14)°.

## Related literature

For the synthesis, see: Huang *et al.* (2004 ▶). For a similar structure, see: Aravindan *et al.* (2003 ▶).
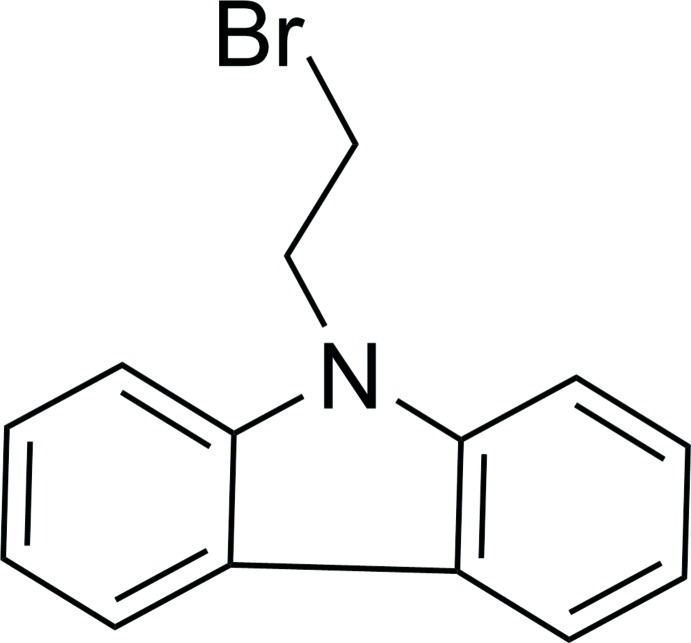


## Experimental

### 

#### Crystal data

C_14_H_12_BrN
*M* *_r_* = 274.16
Monoclinic, 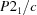

*a* = 5.417 (3) Å
*b* = 12.254 (6) Å
*c* = 17.505 (11) Å
β = 96.46 (3)°
*V* = 1154.6 (11) Å^3^
*Z* = 4
Mo *K*α radiation
μ = 3.53 mm^−1^
*T* = 288 K
0.16 × 0.15 × 0.13 mm

#### Data collection

Rigaku R-AXIS RAPID diffractometer
Absorption correction: multi-scan (*ABSCOR*; Higashi, 1995 ▶) *T*_min_ = 0.599, *T*_max_ = 0.657
10873 measured reflections
2630 independent reflections
2093 reflections with *I* > 2σ(*I*)
*R*_int_ = 0.039

#### Refinement

*R*[*F* ^2^ > 2σ(*F* ^2^)] = 0.032
*wR*(*F* ^2^) = 0.082
*S* = 1.02
2630 reflections
145 parameters
H-atom parameters constrained
Δρ_max_ = 0.23 e Å^−3^
Δρ_min_ = −0.59 e Å^−3^

### 

Data collection: *RAPID-AUTO* (Rigaku, 1998 ▶); cell refinement: *RAPID-AUTO*; data reduction: *CrystalClear* (Rigaku/MSC, 2002 ▶); program(s) used to solve structure: *SHELXS97* (Sheldrick, 2008 ▶); program(s) used to refine structure: *SHELXL97* (Sheldrick, 2008 ▶); molecular graphics: *SHELXTL* (Sheldrick, 2008 ▶); software used to prepare material for publication: *SHELXL97*.

## Supplementary Material

Crystal structure: contains datablock(s) global, I. DOI: 10.1107/S1600536812024397/ng5265sup1.cif

Structure factors: contains datablock(s) I. DOI: 10.1107/S1600536812024397/ng5265Isup2.hkl

Supplementary material file. DOI: 10.1107/S1600536812024397/ng5265Isup3.cml

Additional supplementary materials:  crystallographic information; 3D view; checkCIF report

## Figures and Tables

**Fig. 1.**
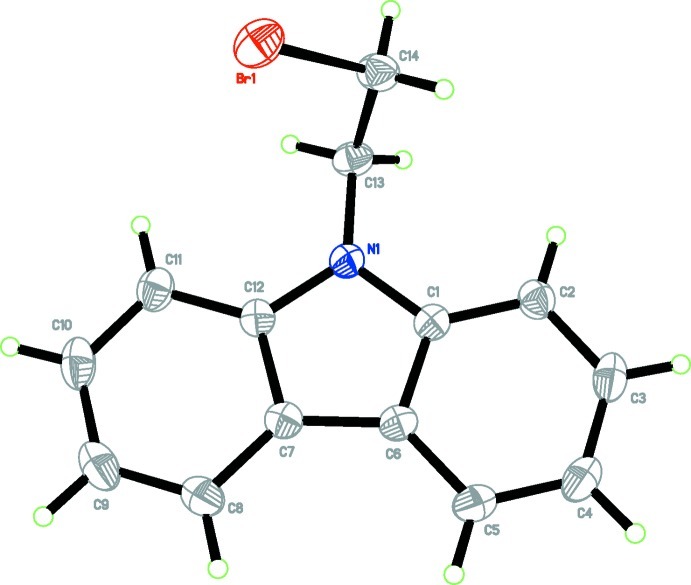
Molecular structure of the title compound with displacement ellipsoids drawn at the 30% probalility level.

## supplementary crystallographic information

### Comment

Carozole and its derivatives are an important type of nitrogen-containing aromatic heterocyclic compounds. These special structure of carbazole compounds endow their distinct various functions as well as wide potential applications. In this paper, we report the crystal structure of the title compound.

The molecular structure of tiltle compound, C_14_H_12_BrN, as shown in Fig.1, all bond lengths and angles are in the normal ranges and are comparable with a reported compounnd (Aravindan *et al.* 2003). The dihedral angel of the two benzene rings is 3.41 (14) °. Van der Waals forces stablize the crystal structure.

### Experimental

The title compound was prepared according to the literature (Huang *et al.* 2004). Single crystals suitable were prepared by slow evaporation of a dichloromethane solution of the compoundat room temperature.

### Refinement

Carbon-bound H-atoms were placed in calculated positions (C—H 0.93 and 0.97 Å) and were included in the refinement in the riding model with *U*_iso_(H) = 1.2 *U*_eq_(C). The (-2 5 5) was omitted owing to bad disagreement.

### Crystal data

**Table:**

C_14_H_12_BrN	*F*(000) = 552
*M**_r_* = 274.16	*D*_x_ = 1.577 Mg m^−^^3^
Monoclinic, *P*2_1_/*c*	Mo *K*α radiation, λ = 0.71073 Å
Hall symbol: -P 2ybc	Cell parameters from 8078 reflections
*a* = 5.417 (3) Å	θ = 3.3–27.5°
*b* = 12.254 (6) Å	µ = 3.53 mm^−^^1^
*c* = 17.505 (11) Å	*T* = 288 K
β = 96.46 (3)°	Block, colorless
*V* = 1154.6 (11) Å^3^	0.16 × 0.15 × 0.13 mm
*Z* = 4	

### Data collection

**Table:**

Rigaku R-AXIS RAPID diffractometer	2630 independent reflections
Radiation source: fine-focus sealed tube	2093 reflections with *I* > 2σ(*I*)
Graphite monochromator	*R*_int_ = 0.039
ω scans	θ_max_ = 27.5°, θ_min_ = 3.3°
Absorption correction: multi-scan (*ABSCOR*; Higashi, 1995)	*h* = −6→7
*T*_min_ = 0.599, *T*_max_ = 0.657	*k* = −15→15
10873 measured reflections	*l* = −22→22

### Refinement

**Table:**

Refinement on *F*^2^	Primary atom site location: structure-invariant direct methods
Least-squares matrix: full	Secondary atom site location: difference Fourier map
*R*[*F*^2^ > 2σ(*F*^2^)] = 0.032	Hydrogen site location: inferred from neighbouring sites
*wR*(*F*^2^) = 0.082	H-atom parameters constrained
*S* = 1.02	*w* = 1/[σ^2^(*F*_o_^2^) + (0.0394*P*)^2^ + 0.3164*P*] where *P* = (*F*_o_^2^ + 2*F*_c_^2^)/3
2630 reflections	(Δ/σ)_max_ = 0.007
145 parameters	Δρ_max_ = 0.23 e Å^−^^3^
0 restraints	Δρ_min_ = −0.59 e Å^−^^3^

### Special details

**Table:**

Experimental. (See detailed section in the paper)
Geometry. All esds (except the esd in the dihedral angle between two l.s. planes) are estimated using the full covariance matrix. The cell esds are taken into account individually in the estimation of esds in distances, angles and torsion angles; correlations between esds in cell parameters are only used when they are defined by crystal symmetry. An approximate (isotropic) treatment of cell esds is used for estimating esds involving l.s. planes.
Refinement. Refinement of F^2^ against ALL reflections. The weighted R-factor wR and goodness of fit S are based on F^2^, conventional R-factors R are based on F, with F set to zero for negative F^2^. The threshold expression of F^2^ > 2sigma(F^2^) is used only for calculating R-factors(gt) etc. and is not relevant to the choice of reflections for refinement. R-factors based on F^2^ are statistically about twice as large as those based on F, and R- factors based on ALL data will be even larger.

### Fractional atomic coordinates and isotropic or equivalent isotropic displacement parameters (Å^2^)

**Table:**

	*x*	*y*	*z*	*U*_iso_*/*U*_eq_	
Br1	0.25417 (5)	1.06970 (2)	0.430911 (16)	0.05611 (12)	
C1	0.0265 (4)	0.88648 (17)	0.22679 (12)	0.0350 (4)	
C2	−0.1470 (4)	0.94431 (18)	0.17824 (14)	0.0420 (5)	
H2	−0.2717	0.9847	0.1974	0.050*	
C3	−0.1273 (5)	0.9395 (2)	0.10030 (14)	0.0506 (6)	
H3	−0.2424	0.9766	0.0664	0.061*	
C4	0.0610 (5)	0.8802 (2)	0.07141 (14)	0.0504 (6)	
H4	0.0706	0.8793	0.0187	0.060*	
C5	0.2328 (4)	0.82325 (19)	0.11943 (13)	0.0446 (5)	
H5	0.3591	0.7846	0.0997	0.054*	
C6	0.2147 (4)	0.82425 (16)	0.19839 (12)	0.0356 (4)	
C7	0.3499 (4)	0.77144 (17)	0.26390 (13)	0.0365 (5)	
C8	0.5493 (4)	0.69878 (19)	0.27313 (15)	0.0473 (6)	
H8	0.6248	0.6755	0.2309	0.057*	
C9	0.6322 (5)	0.6623 (2)	0.34564 (18)	0.0572 (7)	
H9	0.7654	0.6140	0.3522	0.069*	
C10	0.5209 (5)	0.6961 (2)	0.40933 (16)	0.0546 (6)	
H10	0.5813	0.6698	0.4577	0.066*	
C11	0.3225 (4)	0.76790 (19)	0.40274 (14)	0.0465 (5)	
H11	0.2484	0.7903	0.4455	0.056*	
C12	0.2387 (4)	0.80512 (17)	0.32906 (13)	0.0357 (4)	
C13	−0.1196 (4)	0.92796 (18)	0.35461 (14)	0.0417 (5)	
H13A	−0.1073	0.8900	0.4035	0.050*	
H13B	−0.2893	0.9207	0.3308	0.050*	
C14	−0.0643 (4)	1.04724 (19)	0.36924 (15)	0.0466 (5)	
H14A	−0.1951	1.0790	0.3956	0.056*	
H14B	−0.0631	1.0846	0.3204	0.056*	
N1	0.0445 (3)	0.87583 (15)	0.30599 (10)	0.0369 (4)	

### Atomic displacement parameters (Å^2^)

**Table:**

	*U*^11^	*U*^22^	*U*^33^	*U*^12^	*U*^13^	*U*^23^
Br1	0.05008 (16)	0.06487 (19)	0.05276 (18)	−0.01404 (11)	0.00307 (12)	−0.00812 (12)
C1	0.0349 (10)	0.0342 (10)	0.0358 (11)	−0.0048 (8)	0.0038 (8)	−0.0010 (9)
C2	0.0387 (11)	0.0423 (12)	0.0441 (13)	0.0013 (9)	0.0002 (10)	0.0007 (10)
C3	0.0585 (14)	0.0479 (13)	0.0417 (14)	−0.0059 (11)	−0.0106 (11)	0.0088 (10)
C4	0.0685 (16)	0.0497 (13)	0.0322 (12)	−0.0103 (12)	0.0017 (11)	0.0014 (10)
C5	0.0533 (13)	0.0427 (12)	0.0394 (13)	−0.0039 (10)	0.0117 (10)	−0.0061 (10)
C6	0.0370 (10)	0.0337 (10)	0.0363 (11)	−0.0056 (8)	0.0055 (9)	−0.0034 (9)
C7	0.0352 (10)	0.0339 (10)	0.0401 (12)	−0.0040 (9)	0.0035 (9)	−0.0024 (9)
C8	0.0413 (11)	0.0414 (12)	0.0589 (16)	0.0033 (10)	0.0042 (11)	−0.0067 (11)
C9	0.0480 (13)	0.0421 (13)	0.078 (2)	0.0077 (11)	−0.0076 (13)	0.0035 (13)
C10	0.0585 (14)	0.0464 (13)	0.0546 (16)	−0.0029 (12)	−0.0121 (12)	0.0163 (12)
C11	0.0520 (12)	0.0456 (12)	0.0411 (13)	−0.0052 (11)	0.0011 (11)	0.0075 (10)
C12	0.0343 (10)	0.0342 (10)	0.0379 (11)	−0.0038 (8)	0.0018 (8)	0.0014 (9)
C13	0.0355 (10)	0.0477 (13)	0.0432 (13)	−0.0033 (9)	0.0105 (9)	−0.0080 (10)
C14	0.0392 (11)	0.0496 (13)	0.0507 (15)	0.0037 (10)	0.0044 (10)	−0.0084 (11)
N1	0.0376 (9)	0.0403 (9)	0.0329 (9)	0.0028 (8)	0.0048 (7)	−0.0001 (8)

### Geometric parameters (Å, º)

**Table:**

Br1—C14	1.949 (3)	C8—C9	1.373 (4)
C1—N1	1.385 (3)	C8—H8	0.9300
C1—C2	1.389 (3)	C9—C10	1.389 (4)
C1—C6	1.408 (3)	C9—H9	0.9300
C2—C3	1.382 (4)	C10—C11	1.383 (4)
C2—H2	0.9300	C10—H10	0.9300
C3—C4	1.392 (4)	C11—C12	1.395 (3)
C3—H3	0.9300	C11—H11	0.9300
C4—C5	1.372 (4)	C12—N1	1.387 (3)
C4—H4	0.9300	C13—N1	1.447 (3)
C5—C6	1.397 (3)	C13—C14	1.508 (3)
C5—H5	0.9300	C13—H13A	0.9700
C6—C7	1.443 (3)	C13—H13B	0.9700
C7—C8	1.395 (3)	C14—H14A	0.9700
C7—C12	1.410 (3)	C14—H14B	0.9700
			
N1—C1—C2	128.8 (2)	C10—C9—H9	119.4
N1—C1—C6	109.29 (18)	C11—C10—C9	121.7 (2)
C2—C1—C6	121.8 (2)	C11—C10—H10	119.1
C3—C2—C1	117.4 (2)	C9—C10—H10	119.1
C3—C2—H2	121.3	C10—C11—C12	117.0 (2)
C1—C2—H2	121.3	C10—C11—H11	121.5
C2—C3—C4	121.5 (2)	C12—C11—H11	121.5
C2—C3—H3	119.3	N1—C12—C11	129.1 (2)
C4—C3—H3	119.3	N1—C12—C7	109.03 (19)
C5—C4—C3	121.1 (2)	C11—C12—C7	121.9 (2)
C5—C4—H4	119.5	N1—C13—C14	113.85 (19)
C3—C4—H4	119.5	N1—C13—H13A	108.8
C4—C5—C6	118.9 (2)	C14—C13—H13A	108.8
C4—C5—H5	120.5	N1—C13—H13B	108.8
C6—C5—H5	120.5	C14—C13—H13B	108.8
C5—C6—C1	119.2 (2)	H13A—C13—H13B	107.7
C5—C6—C7	134.2 (2)	C13—C14—Br1	112.18 (16)
C1—C6—C7	106.57 (18)	C13—C14—H14A	109.2
C8—C7—C12	119.3 (2)	Br1—C14—H14A	109.2
C8—C7—C6	134.1 (2)	C13—C14—H14B	109.2
C12—C7—C6	106.66 (18)	Br1—C14—H14B	109.2
C9—C8—C7	118.9 (2)	H14A—C14—H14B	107.9
C9—C8—H8	120.5	C12—N1—C1	108.42 (17)
C7—C8—H8	120.5	C12—N1—C13	126.90 (19)
C8—C9—C10	121.2 (2)	C1—N1—C13	124.65 (18)
C8—C9—H9	119.4		
